# SIRT3: Oncogene and Tumor Suppressor in Cancer

**DOI:** 10.3390/cancers9070090

**Published:** 2017-07-12

**Authors:** Margalida Torrens-Mas, Jordi Oliver, Pilar Roca, Jorge Sastre-Serra

**Affiliations:** 1 Grupo Multidisciplinar de Oncología Traslacional, Institut Universitari d´Investigació en Ciències de la Salut (IUNICS), Universitat de les Illes Balears. Cra de Valldemossa, km 7.5, 07122 Palma, Illes Balears 07122, Spain; lida.torrens@uib.es (M.T.-M.); jordi.oliver@uib.es (J.O.); jorge.sastre@uib.es (J.S.-S.); 2 Ciber Fisiopatología Obesidad y Nutrición (CB06/03) Instituto Salud Carlos III, Madrid 28029, Spain; 3 Instituto de Investigación Sanitaria de Baleares (IdISBa), Hospital Universitario, Son Espases, edificio S. E-07120 Palma, Palma de Mallorca, Illes Balears 07120, Spain

**Keywords:** SIRT3, cancer, ROS, antioxidant enzymes, mitochondria

## Abstract

Sirtuin 3 (SIRT3), the major deacetylase in mitochondria, plays a crucial role in modulating oxygen reactive species (ROS) and limiting the oxidative damage in cellular components. SIRT3 targets different enzymes which regulate mitochondrial metabolism and participate in ROS detoxification, such as the complexes of the respiratory chain, the isocitrate dehydrogenase, or the manganese superoxide dismutase. Thus, SIRT3 activity is essential in maintaining mitochondria homeostasis and has recently received great attention, as it is considered a fidelity protein for mitochondrial function. In some types of cancer, SIRT3 functions as a tumoral promoter, since it keeps ROS levels under a certain threshold compatible with cell viability and proliferation. On the contrary, other studies describe SIRT3 as a tumoral suppressor, as SIRT3 could trigger cell death under stress conditions. Thus, SIRT3 could have a dual role in cancer. In this regard, modulation of SIRT3 activity could be a new target to develop more personalized therapies against cancer.

## 1. Introduction

Sirtuins are a family of NAD^+^-dependent deacetylases which regulate signaling pathways involved in cellular proliferation and differentiation, metabolism, response to stress, and cancer [1,2]. Seven isoforms have been described in mammals, and sirtuins 3, 4, and 5 are exclusively located in the mitochondria [3,4,5]. Among these, sirtuin 3 (SIRT3) has recently gained great attention, as it presents the most robust mitochondrial deacetylase activity and targets key proteins for the proper function and metabolism of these organelles [6].

SIRT3 is synthesized as a 44 kDa peptide with an N-terminal sequence as a targeting signal for mitochondrial localization. Under stress conditions, SIRT3 translocates to the mitochondrial matrix, where is activated by a protease, which yield the 28-kDa active form of SIRT3 [7,8,9]. Recent studies point out a possible nuclear localization of SIRT3 in the nucleus, where it may regulate gene expression in response to stress [7,10].

## 2. SIRT3 as a Mitochondrial Fidelity Protein

Mitochondria are crucial organelles for several cellular processes, such as energy production through oxidative phosphorylation, cell cycle and proliferation, apoptosis, etc. [2,11]. Nevertheless, the mitochondrial respiratory chain is responsible for the production of reactive oxygen species or oxygen reactive species (ROS) [12]. When ROS production is controlled and ROS levels are under a certain threshold, they act as second messengers and stimulate cellular proliferation. However, high ROS levels can be harmful to the cell, damaging proteins, lipids, and DNA, thus contributing to mitochondrial dysfunction and carcinogenesis [13,14,15,16].

For this reason, fidelity proteins are essential for the maintenance and proper function of mitochondria [17,18,19]. Around 35% of mitochondrial proteins are estimated to be regulated by acetylation, and the majority of them are involved in energy metabolism. This observation suggests that acetylation may be a critical post-translational modification in these organelles [11,20,21].

**Figure 1:**
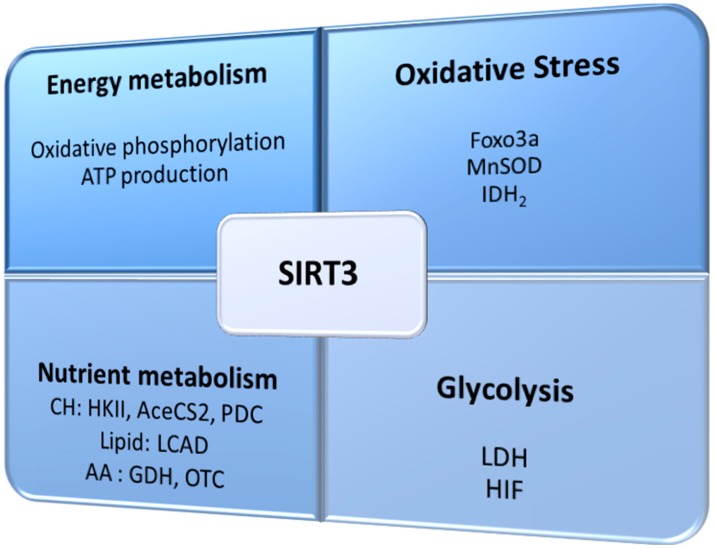
Sirtuin 3 (SIRT3) is involved in several processes, including metabolism and oxidative stress, which can be crucial for tumor development and promotion. Foxo3a: forkhead box O3; MnSOD: manganese superoxide dismutase; IDH2: isocitrate dehydrogenase; CH: carbohydrate; HKII: hexokinase II; AceCS2: acetyl-CoA synthase 2; PDC: pyruvate dehydrogenase complex; LCAD: long chain acyl-CoA dehydrogenase; GDH: glutamate dehydrogenase; OTC: ornithine transcarbamylase; LDH: lactate dehydrogenase; HIF: hypoxia-inducible factor.

SIRT3 has been reported to deacetylate and activate proteins that are essential against oxidative stress, including antioxidant enzymes and enzymes involved in mitochondrial function and ATP synthesis [22,23,24]. Loss of SIRT3 function is linked to the development of a permissive phenotype for carcinogenesis, as mitochondrial proteins become hyperacetylated and cells show elevated oxidative stress, which leads to mitochondrial dysfunction [5,11]. Furthermore, some studies in tumors report a correlation between SIRT3 expression and the evolution of several types of cancer [25,26,27,28,29,30,31]. As a stress-responsive protein, SIRT3 function is to regulate ROS production to avoid damage to cellular components [5,32]. Thus, SIRT3 is crucial in the maintenance of mitochondrial function and integrity of these organelles, and has been referred to as the guardian of the mitochondria, similarly to the consideration of p53 as the guardian of the genome [33]. The main processes in which SIRT3 takes part, directly and indirectly, are schematized in Figure 1.

## 3. SIRT3 Regulates Oxidative Stress and Cellular Metabolism

As mentioned above, SIRT3 is key for the mitochondrial integrity and proper function under stress conditions [2,34]. Several studies show that mice lacking SIRT3 show no important affections under basal conditions, although after fasting, exercise or calorie restriction these mice develop several diseases, including some types of cancer. These conditions have an increase in ROS production in common that leads to oxidative stress, which is proposed to be the cause of disease [18,35,36].

SIRT3 limits ROS production as well as reduces the resulting oxidative damage. In this regard, SIRT3 is considered a key ROS scavenger in the cell, since it targets two main oxidative stress-responsive proteins: manganese superoxide dismutase (MnSOD) and isocitrate dehydrogenase (IDH) [37,38,39,40]. MnSOD is a mitochondrial antioxidant enzyme crucial for the cell, as it converts highly reactive superoxide ions into hydrogen peroxide, which is later neutralized to water by catalase and other mitochondrial peroxidases [38,41,42]. On the other hand, deacetylation of IDH increases its activity, stimulating the conversion of isocitrate to α-ketoglutarate. This reaction generates nicotinamide adenine dinucleotide phosphate (NADPH), which is required for the regeneration of reduced glutathione, another main antioxidant of the cell [43,44,45]. Some recent studies also indicate that SIRT3 could affect gene expression of these antioxidant enzymes, presumably through deacetylation and activation of transcriptional factor FoxO3a, which regulates essential proteins for mitochondrial homeostasis such as MnSOD and catalase [39,44,46,47,48].

SIRT3 also modulates energy metabolism by directly deacetylating and activating several enzymes involved in regulation of mitochondrial metabolism, including the Krebs cycle, the respiratory chain and the oxidation of fatty acids [6,49], contributing to proper mitochondrial function. This way, SIRT3 induces higher oxidative phosphorylation and ATP production [50].

For instance, SIRT3 deacetylates and inactivates cyclophilin D, which results in the detachment of hexokinase II from mitochondria, thereby inhibiting glycolysis [51]. Furthermore, SIRT3 can increase acetyl-CoA availability for the tricarboxylic acid cycle (TCA) in mitochondria activating acetyl-CoA synthase 2 (AceCS2) [52,53] and some activator enzymes of the pyruvate dehydrogenase complex (PDC) [54]. A recent study suggests that SIRT3 could also deacetylate and enhance the activity of lactate dehydrogenase (LDH), promoting anaerobic glycolysis [55].

SIRT3 also participates in the regulation of lipid metabolism through deacetylation and activation of long chain acyl-CoA dehydrogenase (LCAD), thus stimulating fatty acid oxidation [56]. It has also been described that SIRT3 enhances the synthesis of β-hydroxybutyrate by activating 2-hydroxy-3-methylglutaryl CoA synthase 2 [57].

Glutamate dehydrogenase (GDH) is another promising target of SIRT3. GDH is involved in amino acid metabolism, driving them into the Krebs cycle to be used as fuel, and is affected in tumoral cells, contributing to its survival and proliferation [37,58]. SIRT3 can also promote the urea cycle by deacetylating and activating ornithine transcarbamylase (OTC) under energy restriction [59].

SIRT3 also takes part in the coordination of the TCA cycle, the electron transport chain (ETC) and ATP synthesis through deacetylation and activation of different complexes of the oxidative phosphorylation [50]. For instance, it has been reported that SIRT3 deacetylates and activates different subunits of all ETC complexes [50,60,61,62,63] and ATPase [64], thus enhancing mitochondrial function. Furthermore, SIRT3 activates liver kinase B1 (LKB1), which in turn activates AMP-activated protein kinase (AMPK), leading to ATP synthesis [65].

SIRT3 is involved in mitochondrial biogenesis and mitochondrial dynamics. Under stress conditions, the peroxisome proliferator-activated receptor γ co-activator 1-α (PGC-1α) along with the estrogen receptor-related alpha (ERRα), involved in mitochondrial biogenesis, can regulate expression and protein levels of SIRT3 [66,67]. On the other hand, SIRT3 indirectly participates in PGC1-α expression through activating AMPK signaling pathway, which results in cAMP response element-binding protein (CREB) phosphorylation and increased PGC1-α gene expression [68]. The establishment of this positive feedback loop is crucial for the regulation of mitochondrial biogenesis and function, activating mitochondrial enzymes involved in antioxidant defenses and metabolism [63,66,68]. Finally, SIRT3 also deacetylates and activates optic atrophy 1 (OPA1), which is involved in mitochondrial fusion [69].

SIRT3 also contributes to mitochondrial quality control. For instance, SIRT3 has been described to coordinate the mitochondrial unfolded protein response and upregulate mitophagy machinery [70]. Furthermore, SIRT3 can suppress the formation and opening of the mitochondrial permeability transition pore, preventing mitochondrial dysfunction [71]. SIRT3 also deacetylates 8-oxoguanine glycosylase 1 (OGG1), which prevents its degradation. This way, OGG1 contributes to mitochondrial DNA repair, protecting integrity of mitochondria, and preventing apoptosis [72].

**Figure 2:**
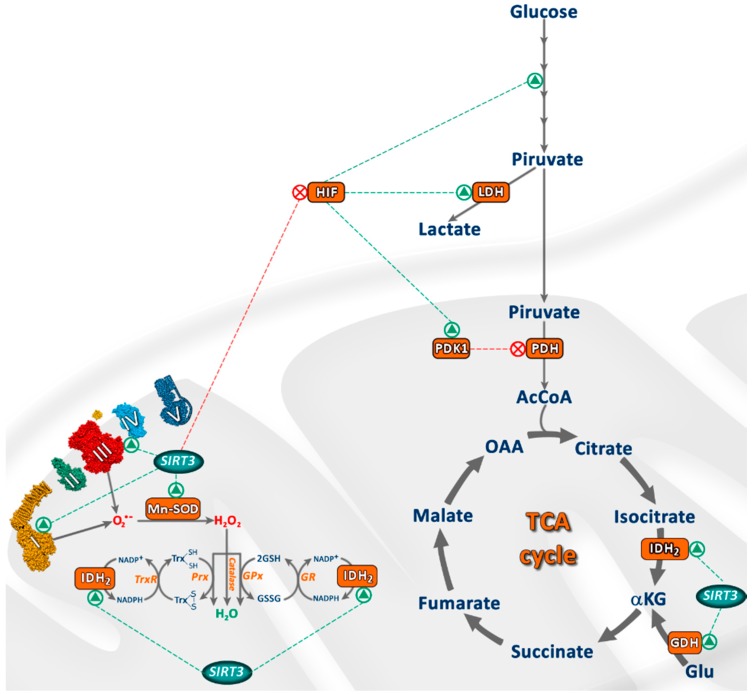
Main targets of SIRT3 in mitochondria. Green triangles represent protein activation, and red crosses represent target inactivation.

These observations indicate the role of SIRT3 in energy metabolism, ATP synthesis, mitochondrial function and mitochondrial ROS scavenging, which are crucial for the proper function of mitochondria. The main targets of SIRT3 are shown in Figure 2.

Metabolism can be crucial in cancer development and progression, as tumoral cells undergo a metabolic reprogramming to meet energy demands for continued growth and proliferation, known as the Warburg effect [73,74]. For this reason, cancer cells show a shift from oxidative phosphorylation to glycolysis as the main metabolic process to obtain ATP [55,75,76]. Apart from a rapid energy supply, cells also obtain different intermediates to meet the requirements for the synthesis of macromolecules and sustain cell division [77,78].

SIRT3 plays a pivotal role in this metabolic reprogramming through regulation of all the targets mentioned before. Moreover, SIRT3 leads to destabilization and inactivation of hypoxia inducible factor 1-alpha (HIF-1 α). This nuclear factor induces expression of genes involved in glycolysis, directly promoting the Warburg effect, and genes involved in angiogenesis, contributing to tumor evolution [1,35,36]. Thus, loss of SIRT3 is associated to high oxidative stress and ROS production, as well as to metabolic reprogramming, which contributes to carcinogenesis.

## 4. SIRT3 Is Involved in Proliferation and Apoptosis Pathways

Besides its function in metabolism regulation, SIRT3 has been referred to as an oncogene in some types of cancer, as SIRT3 can maintain ROS production at the appropriate levels to prevent apoptosis and promote cell proliferation [18,33,70]. Furthermore, SIRT3 regulates mitochondrial homeostasis and preserves mitochondrial membrane integrity, thus increasing cellular resistance to oxidative stress [24,51,72]. In accordance to this proliferative function, in cervical cancer cells SIRT3 has been reported to interact with the protein Ku70, involved in DNA repair, which results in apoptosis avoidance under stress [79]. Additionally, p53 has been recently described as a target of SIRT3. In bladder cancer, deacetylation of p53 induces cellular proliferation, rescuing cells from growth arrest caused by p53 [80]. Some reports suggest that SIRT3 could have a crucial role in the development and progression of some types of cancer acting as a tumour promoter, such as breast cancer [46], colon cancer [30], gastric cancer [55], esophageal cancer [81], oral squamous cell carcinoma [82], melanoma [83], and renal cancer [58]. These studies show a correlation between high SIRT3 expression and poorer clinical prognosis. Furthermore, SIRT3 knockdown sensitizes cancer cells to cytotoxic treatments [46,82] and reduces cell proliferation [46,82,83].

On the contrary, some studies suggest a tumour suppressor role for SIRT3. It has been described that SIRT3 induces cell arrest and apoptosis, by regulating proteins such as Bcl-2, p53 of HIF-1α [35,80,84,85]. In agreement with this protective function, mice lacking SIRT3 develop tumors more quickly than control mice. Additionally, tumors lacking SIRT3 grow faster and show greater volume than control tumors in xenograft models [18,35]. SIRT3 has been described as a tumor suppressor in breast cancer [25], hepatocellular carcinoma [86,87,88], B cell malignancies and leukemia [85,89], and metastatic ovarian cancer [90]. In these reports, SIRT3 expression correlates with a good outcome and a general increase in overall survival of cancer patients. The mechanisms described include inhibition of proliferation when SIRT3 is overexpressed [86,89,91], and limiting metabolic reprogramming [51,89].

## 5. Conclusions

As it has been discussed throughout this review, SIRT3 seems to prevent cell death under oxidative stress, while other studies report a pro-apoptotic function for this deacetylase. Therefore, SIRT3 shows a dual role in cancer, as it can act as a tumour suppressor or a tumour promoter, depending on the cellular context. Cancer cells usually show higher ROS levels than normal cells, which confers advantages in tumor promotion and progression, as well as resistance to chemotherapy. However, some anticancer therapies are based on its ability to further increase ROS production to reach toxic levels, resulting in cell death and overcoming treatment resistance [92,93].

For this reason, SIRT3 rises as a possible target to develop new therapeutic strategies against cancer. For instance, in breast cancer SIRT3 could confer resistance to tamoxifen, a commonly used therapy to block the estrogen receptor in this type of cancer [29]. In this way, some studies report that SIRT3 silencing results in a decreased cellular proliferation and induces cell death, hence it could improve chemotherapy efficacy for some cancers [4,46,70]. Therefore, modulation of SIRT3 activity could be an approach to improve therapies against cancer, especially to overcome acquired resistance to treatment.
